# Teaching good infection control practices with fun: impact of the serious game Flu.0

**DOI:** 10.1186/2047-2994-4-S1-I10

**Published:** 2015-06-16

**Authors:** A-G Venier, S Marie, T Duroux, C Bervas, P Parneix

**Affiliations:** 1 CCLIN SUD-OUEST, Bordeaux, France; 2 ARLIN Limousin, Limoges, France

## Introduction

Flu outbreaks usually reveal that practices and knowledge about the diagnosis of influenza, its treatment and infection control measures must be improved. A serious game, “Flu.0”, was created to teach the 8 key points to know and do when facing one or more patient infected by influenza.

## Objectives

To evaluate the impact of playing to ‘Flu.0’ on the knowledge and practices of nurses and physicians.

## Methods

Flu.0 is free and can be played online or downloaded; A call for participation to play and evaluate the game was performed. Players were asked to complete a questionnaire before and after the game to give their opinion on sentences about flu, to write what they learned with the game and the main thing they would do differently. A descriptive analysis was performed and the evolution of the answers was analysed.

## Results

Physicians were 264 to participate (including 213 fellows), senior nurses 62 and nurses students 577; 95% learnt at least something. The main knowledge acquired was about rapid test for influenza (32%) and additional precautions (19%). Significantly, players agreed more after the game that seasonal flu is a not benign disease, that flu vaccination of health care workers is useful, knew more about antiviral treatment and felt better prepared to face a flu case (p<0.001). Thanks to the game 47% of physician/senior nurses and 80% of nurses students declared they would perform better additional precautions.

## Conclusion

A serious game is an innovative quick and efficient tool for infection control team to improve patient safety.

## Disclosure of interest

None declared.

